# Sociodemographic Factors and Access to Digital Resources Among Medicare Beneficiaries in Nonmetropolitan Areas

**DOI:** 10.1093/geroni/igaf122.4202

**Published:** 2025-12-31

**Authors:** Brian Nguyen, Boon Peng Ng

**Affiliations:** University of Central Florida, Orlando, Florida, United States; University of Central Florida, Orlando, Florida, United States

## Abstract

The COVID-19 pandemic accelerated telehealth adoption, but disparities in digital access may hinder its potential, especially for older adults in rural areas. This cross-sectional study examined sociodemographic factors of digital access using 2022 Medicare Current Beneficiary Survey Public Use File. The sample included 1,732 Medicare beneficiaries aged ≥65 living in nonmetropolitan areas. The dependent variable of digital access was categorized as: (1) access to both a computer/tablet and the internet, (2) access to either, and (3) access to neither (reference group). A survey-weighted multinomial logit model was conducted to assess associations between sociodemographic factors and digital access. Overall, 71.7% of nonmetro beneficiaries had both computer and internet access, 14.4% had one or the other, and 13.9% had neither. Beneficiaries aged ≥75 (vs 65–74) had significantly lower odds of having both computer and internet access (OR = 0.30, p < 0.001) and access to either (OR = 0.39, p = 0.003). Males had lower odds of having both access (OR = 0.64, p = 0.023). Non-Hispanic Blacks (NHB) and Other minorities had lower odds of having both access (for NHB, OR = 0.45, p < 0.001). Beneficiaries with lower education (vs more than a high school education) had lower odds of having both access (for less than a high school education, OR = 0.13, p < 0.001). Income under $25,000 was associated with lower odds of having both access (OR = 0.36, p < 0.001). Older age, male sex, minority race/ethnicity, lower education, and lower income are key factors of reduced digital access among nonmetro Medicare beneficiaries, underscoring the need for targeted interventions and policies to expand telehealth access for these at-risk populations.

